# Association of *ADH1B* Arg47His polymorphism with the risk of cancer: a meta-analysis

**DOI:** 10.1042/BSR20181915

**Published:** 2019-04-02

**Authors:** Boyu Tan, Ning Ning

**Affiliations:** 1 Department of Pharmacy, First Affiliated Hospital of Hunan Normal University, Hunan Provincial People’s Hospital, Changsha 410005, Hunan, China; 2 Department of Medicine, First Affiliated Hospital of Hunan Normal University, Hunan Provincial People’s Hospital, Changsha 410005, Hunan, China

**Keywords:** ADH1B, cancer, meta-analysis, polymorphism, risk

## Abstract

Alcohol consumption has been established to be a major factor in the development and progress of cancer. Genetic polymorphisms of alcohol-metabolism genes result in differences between individuals in exposure to acetaldehyde, leading to possible carcinogenic effects. Arg47His (rs1229984 G > A) in *ADH1B* have been frequently studied for its potential effect on carcinogenesis. However, the findings are as yet inconclusive. To gain a more precise estimate of this potential association, we conducted a meta-analysis including 66 studies from 64 articles with 31999 cases and 50964 controls. The pooled results indicated that *ADH1B* Arg47His polymorphism is significantly associated with the decreased risk of overall cancer (homozygous model, odds ratio (OR) = 0.62, 95% confidence interval (CI) = 0.49–0.77; heterozygous model, OR = 0.71, 95% CI = 0.60–0.84; recessive model, OR = 0.83, 95% CI = 0.76–0.91; dominant model, OR = 0.62, 95% CI = 0.53–0.72; and allele comparison, OR = 0.82, 95% CI = 0.75–0.89). Stratified analysis by cancer type and ethnicity showed that a decreased risk was associated with esophageal cancer and head and neck cancer amongst Asians. In conclusion, our meta-analysis suggested that *ADH1B* Arg47His polymorphism was significantly associated with decreased overall cancer risk. These findings need further validation in large multicenter investigations.

## Introduction

Cancer is a major public health problem worldwide. According to GLOBOCAN worldwide estimates, an estimated 14.1 million new cancer cases and 8.2 million cancer-related deaths occurred in 2012 [1]. In addition, the incidence of cancer is predicted to reach 25 million worldwide by 2032 [2]. This growing cancer burden is expected as populations expand and age. Meanwhile, certain lifestyles, such as alcohol consumption, are likely to further boost the burden [1–3].

Alcohol consumption is the third-largest risk factor for global health burden [4]. Approximately 3.3 million deaths, almost 5.9% of total deaths worldwide in 2012, were attributable to alcohol consumption [5]. As early as 2002, approximately 3.6% of all cancers and 3.5% of all cancer deaths were reported due to alcohol consumption [3]. It is well established that alcohol is first catalytically oxidized to acetaldehyde, mainly by alcohol dehydrogenases (ADH), and then to harmless acetate by aldehyde dehydrogenases (ALDH) [6,7]. Acetaldehyde may stimulate carcinogenesis by disrupting DNA synthesis and repair, inhibiting DNA methylation, and by interacting with retinoid metabolism [8,9]. Genetic polymorphisms of alcohol-metabolism genes result in differences between individuals in exposure to acetaldehyde, leading to possible carcinogenic effects [10]. Amongst them, Arg47His (rs1229984 G > A) in *ADH1B* have been frequently studied for its potential effect on the carcinogenesis. Compared with the Arg/Arg individuals, the His/His individuals have a 40-fold higher enzyme activity oxidized alcohol to toxic acetaldehyde [7].

Epidemiologic studies have extensively explored the association between *ADH1B* Arg47His polymorphism and cancer risk. However, the findings are as yet inconclusive. Several meta-analyses published before 2016 associated this polymorphism only with esophageal, head and neck, gastric, colorectal, and upper aerodigestive tract cancer [11–16]. However, no meta-analyses have ever investigated the association between *ADH1B* Arg47His polymorphism and overall cancer risk, including other types of cancer. In addition, several more studies with larger sample size were published since 2016 [17–24]. Therefore, we performed an updated meta-analysis including the most recent and relevant studies to clarify the association between *ADH1B* Arg47His polymorphism and the overall cancer risk, involving 66 studies with 31999 cases and 50964 controls [17–80].

## Methods

### Identification of relevant studies

A systematic literature search was conducted in the following electronic databases: Medline and Embase database up to 1 July 2018. The following search terms were used: ‘ADH1B or ADH2’ or ‘polymorphism or variant’ or ‘cancer or carcinoma or tumor’. In addition, reviews and references lists of eligible studies were manually searched to identify additional relevant articles.

### Inclusion and exclusion criteria

The eligible articles must meet the following criteria. The inclusion criteria were as follows: (i) studies evaluating the association between *ADH1B* Arg47His polymorphism and overall cancer risk; (ii) case–control studies; (iii) studies with sufficient information to calculate the odds ratio (OR) and its 95% confidence interval (CI). The major exclusion criteria were as follows: (i) no control group; (ii) duplicate publication; (iii) reviews, meta-analyses, conference reports, or editorial articles; (iv) no available data.

### Data extraction

Investigators independently extracted the relevant information from all eligible studies according to the inclusion and exclusion criteria listed above. A final consensus was achieved regarding each selected study. The following information was extracted from each study: first author’s surname, publication year, country, ethnicity, cancer type, control source, genotyping method, number of cases and controls with different genotypes, and Hardy–Weinberg equilibrium (HWE) of genotypes in controls.

### Statistical analysis

The strength of the association between *ADH1B* Arg47His polymorphism and overall cancer risk was evaluated by calculating ORs and 95% CIs. The pooled ORs were also estimated using homozygous model (His/His vs. Arg/Arg), heterozygous model (Arg/His vs. Arg/Arg), recessive model [His/His vs. (Arg/His + Arg/Arg)], dominant model [(Arg/His + His/His) vs. Arg/Arg], as well as allele comparison (His vs. Arg). Stratification analyses were further conducted according to ethnicity, cancer type, control source, and HWE. Chi square-based Q-test was applied to assess between-study heterogeneity. If no heterogeneity (*P*>0.10) was found, the fixed-effect model (Mantel–Haenszel method) was performed [81]. Otherwise, the random-effect model (DerSimonian and Laird method) was used [82]. Sensitivity analysis was carried out to assess the stability of the results, and potential publication bias was assessed with Begg’s funnel plot and Egger’s linear regression test [83]. All the statistical analyses were calculated using STATA software (version 11.0, Stata Corporation, College Station, TX). A *P*-value less than 0.05 was considered statistically significant.

## Results

### Study characteristics

**Figure 1:**
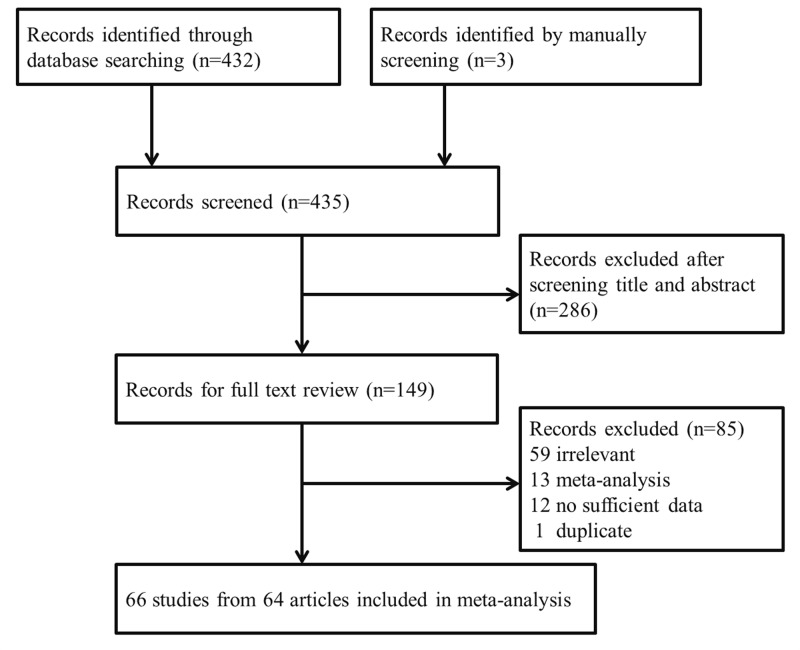
Flow chart of studies included in our meta-analysis

As listed in Figure 1, a total of 432 potential records were initially identified from Medline and Embase using the search terms listed above. After a screening of the titles and abstracts, 146 publications were subjected for further evaluation. Of them, 59 articles were excluded for irrelevant information, 13 for only meta-analysis, 12 for no sufficient data, and 1 was excluded for duplicate study. In addition, three studies were manually identified from reviews and references lists of the eligible studies. Ultimately, 64 articles investigating the association between *ADH1B* Arg47His polymorphism and cancer risk were included in the final meta-analysis [17–80].

Overall, 66 studies from 64 articles with 31999 cases and 50964 controls were finally included in our meta-analysis. As shown in Table 1, there were 48 studies conducted amongst Asians, 15 amongst Caucasians, and 3 amongst mixed ethnic group. With respect to cancer type, 23 studies addressed esophageal cancer, 16 head and neck cancer, 10 colorectal cancer, 6 gastric cancer, 4 hepatocellular, 3 upper aerodigestive tract cancer, 2 pancreatic and 1 bladder and breast cancer. Regarding control source, 34 studies were hospital-based and 32 studies were population-based. With respect to HWE, 52 met HWE, 5 departed from HWE, and 9 had not enough information.

**Table 1:** Main characteristics of included studies in our meta-analysis

Author	Year	Country	Ethnicity	Cancer type	Control source	Genotyping method	Number of cases			Number of controls			HWE
							GG	GA	AA	GG	GA	AA	
Zhong	2016	China	Asian	Colorectal	HB	PCR-RFLP	85	125	64	152	172	34	Yes
Masaoka	2016	Japan	Asian	Bladder	HB	TaqMan	3	38	33	27	265	448	Yes
Liu	2016	China	Asian	Hepatocellular	HB	Affymetrix	48	262	283	236	1229	1748	Yes
Kagemoto	2016	Japan	Asian	Esophageal	PB	Multiplex PCR	31	36	50	60	389	676	Yes
Chen	2016	China	Asian	Gastric	HB	PCR-RFLP	83	117	46	104	125	45	Yes
Ji	2015	Korea	Asian	Head and neck	HB	TaqMan	26	107	127	15	125	190	Yes
Hidaka	2015	Japan	Asian	Gastric	PB	TaqMan	32	173	252	35	168	254	Yes
Bediaga	2015	Spain	Caucasian	Head and neck	PB	TaqMan	78	6^1^	6^1^	203	39^1^	39 ^1^	NA
Ye	2014	China	Asian	Esophageal	HB	PCR-RFLP	224	400	377	150	578	663	Yes
Tsai	2014	China	Asian	Head and neck	HB	TaqMan	47	165	224	25	221	268	No
Chung	2014	China	Asian	UADT	HB	MassARRAY	68	76	108	25	111	125	Yes
Yuan	2013	China	Asian	Head and neck	PB	TaqMan	42	180	170	72	362	455	Yes
Wu	2013	China	Asian	Esophageal	PB	TaqMan	138	309	355	101	410	510	Yes
Gao	2013	China	Asian	Esophageal	PB	TaqMan	252	907	939	199	909	1155	Yes
Dura	2013	Dutch	Caucasian	Esophageal	PB	TaqMan	326	20	0	406	23	0	Yes
Crous-Bou	2013	Spain	Caucasian	Colorectal	PB	Illumina	457	324	79	513	360	54	Yes
Liang	2012	Island	Mixed	Head and neck	PB	TaqMan	530	38	5	593	76	15	No
Gu	2012	China	Asian	Esophageal	HB	MassArray	53	168	158	26	170	182	Yes
Ferrari	2012	France	Caucasian	Colorectal	PB	TaqMan	1129	97	5	1800	176	6	Yes
Duell	2012	Spain	Caucasian	Gastric	PB	Illumina	317	45	2	1133	132	6	Yes
Chiang	2012	China	Asian	Colorectal	HB	PCR-RFLP	7	34	62	43	205	297	Yes
Yin	2011	Japan	Asian	Colorectal	PB	PCR-RFLP	25	161	268	71	393	588	Yes
Wang	2011	China	Asian	Esophageal	HB	PCR-CTPP	15	34	33	17	67	78	Yes
McKay	2011	France	Caucasian	UADT	PB	Illumina	6776	416^1^	416^1^	7742	907^1^	907 ^1^	NA
Marichalar-Mendia	2011	Spain	Caucasian	Head and neck	PB	TaqMan	80	7^1^	7^1^	203	39^1^	39 ^1^	NA
Ji	2011	Korea	Asian	Head and neck	HB	TaqMan	30	87	108	15	112	174	Yes
Hakenewerth	2011	U.S.A.	Mixed	Head and neck	PB	Illumina	1192	31^1^	31^1^	1243	79^1^	79^1^	NA
Wei	2010	U.S.A.	Caucasian	Head and neck	HB	PCR-RFLP	1059	51	0	1075	52	2	Yes
Tanaka	2010	Japan	Asian	Esophageal	HB	Affymetrix	151	591^1^	591^1^	44	776^1^	776 ^1^	NA
Soucek	2010	Czech	Caucasian	Head and neck	HB	TaqMan	101	21	0	111	10	1	Yes
Mohelnikova- Duchonova	2010	Czech	Caucasian	Pancreatic	PB	TaqMan	213	22	0	242	22	1	Yes
Garcia	2010	Brazil	Mixed	Head and neck	HB	PCR-RFLP	195	12	0	213	29	2	Yes
Cao	2010	China	Asian	Gastric	PB	DHPLC	40	148	194	29	160	193	Yes
Yang	2009	China	Asian	Colorectal	HB	SNPLex	39	181	205	62	319	370	Yes
Oze	2009	Japan	Asian	UADT	HB	TaqMan	71	222	292	53	408	709	Yes
Kawase	2009	Japan	Asian	Breast	HB	TaqMan	25	162	265	47	322	539	Yes
Kanda	2009	Japan	Asian	Pancreatic	HB	TaqMan	4	55	101	74	551	975	Yes
Ding	2009	China	Asian	Esophageal	PB	DHPLC	8	75	108	19	96	106	Yes
Cui	2009	Japan	Asian	Esophageal	PB	Illumina	194	363	510	151	986	1626	Yes
Akbari	2009	Iran	Asian	Esophageal	PB	MassARRAY	21	232	490	73	471	827	Yes
Solomon	2008	India	Asian	Head and neck	HB	PCR-RFLP	13	56	57	8	38	54	Yes
Lee	2008	China	Asian	Esophageal	HB	PCR-RFLP	117	149	140	46	275	335	Yes
Guo	2008	China	Asian	Esophageal	HB	PCR-RFLP	17	25	38	24	168	288	Yes
Gao	2008	China	Asian	Colorectal	PB	DHPLC	15	73	102	20	109	93	Yes
Ding	2008	China	Asian	Hepatocellular	PB	PCR-RFLP	21	132	54	26	97	84	Yes
Zhang	2007	U.S.A.	Caucasian	Gastric	PB	TaqMan	261	31	1	352	48	1	Yes
Yin	2007	Japan	Asian	Colorectal	PB	PCR-RFLP	40	294	345	37	289	452	Yes
Yang	2007	China	Asian	Esophageal	PB	PCR-CTPP	33	80	78	22	76	100	Yes
Hiraki	2007	Japan	Asian	Head and neck	HB	TaqMan	26	75	138	31	213	471	Yes
Asakage	2007	Japan	Asian	Head and neck	PB	PCR-RFLP	31	223	388	19	28	49	No
Sakamoto	2006	Japan	Asian	Hepatocellular	HB	PCR-CTPP	12	73	124	13	103	159	Yes
Matsuo	2006	Japan	Asian	Colorectal	HB	PCR-CTPP	19	102	136	36	259	473	Yes
Hashibe	2006	France	Caucasian	Head and neck	HB	TaqMan	719	47^1^	47^1^	877	108^1^	108 ^1^	NA
Hashibe	2006	France	Caucasian	Esophageal	HB	TaqMan	163	4^1^	4^1^	792	95^1^	95 ^1^	NA
Chen	2006	China	Asian	Esophageal	HB	PCR-RFLP	88	117	125	39	240	313	Yes
Yang	2005	China	Asian	Esophageal	HB	PCR-CTPP	6	85	74	22	168	304	Yes
Wu	2005	China	Asian	Esophageal	PB	PCR-RFLP	39	49	46	16	191	130	No
Landi	2005	France	Caucasian	Colorectal	HB	Millipore	292	54	2	263	48	3	Yes
Risch	2003	Germany	Caucasian	Head and neck	PB	PCR-RFLP	227	18	0	227	24	0	Yes
Chao	2003	China	Asian	Esophageal	HB	PCR-RFLP	19	41	28	7	43	55	Yes
Yokoyama	2002	Japan	Asian	Esophageal	PB	PCR-RFLP	51	73	110	31	220	383	Yes
Boonyaphiphat	2002	Thailand	Asian	Esophageal	HB	APLP	15	86	101	28	139	94	No
Yokoyama	2001	Japan	Asian	Esophageal	PB	PCR-RFLP	56	56^1^	56^1^	145	381^1^	381 ^1^	NA
Yokoyama	2001	Japan	Asian	Gastric	PB	PCR-RFLP	28	10^1^	10^1^	145	381^1^	381 ^1^	NA
Takeshita	2000	Japan	Asian	Hepatocellular	PB	PCR-RFLP	3	36	63	8	43	74	Yes
Hori	1997	Japan	Asian	Esophageal	HB	PCR-RFLP	20	31	40	5	20	43	Yes

Abbreviations: APLP, amplified product length polymorphism; DHPLC, denaturing high-performance liquid chromatography; HB, hospital-based, NA, not applicable; PB, population-based; PCR-CTPP, PCR with the confronting-two-pair primer; PCR-RFLP, PCR-restriction fragment length polymorphism; UADT, upper aerodigestive tract.

1 The number of GA + AA.

### Meta-analysis results

**Figure 2:**
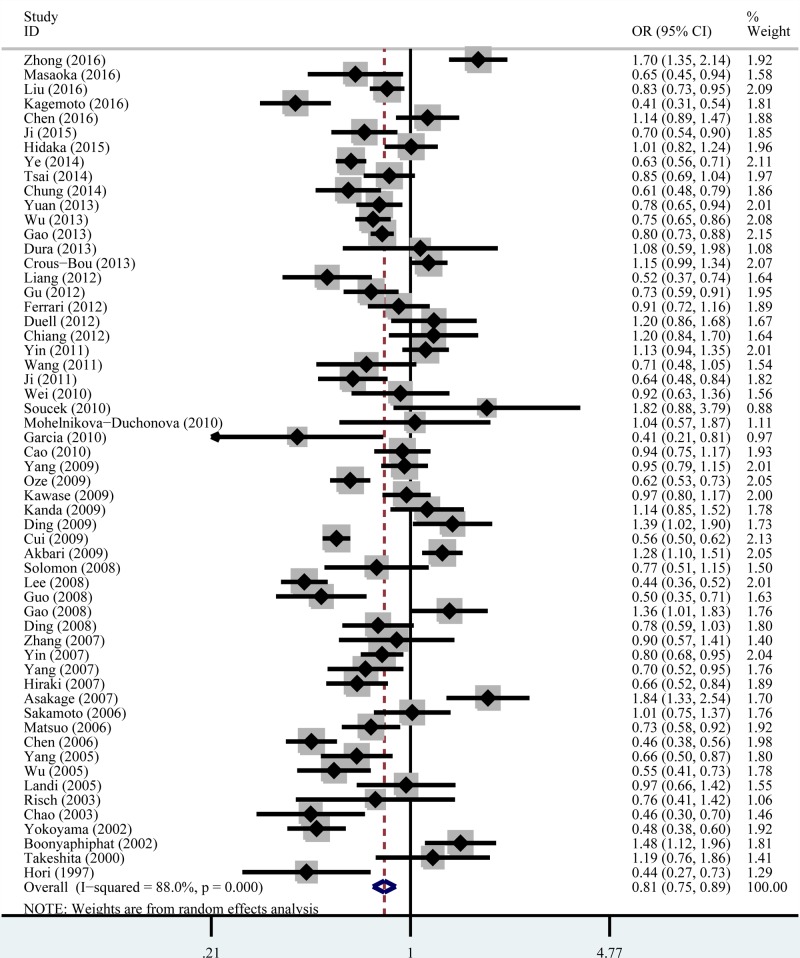
Forest plot of the association between *ADH1B* Arg47His polymorphism and the overall cancer risk under the allele comparison model

The main results for the association between *ADH1B* Arg47His polymorphism and cancer risk are shown in Table 2 and Figure 2. We found that *ADH1B* Arg47His polymorphism significantly associated with the decreased risk of overall cancer under all the five genetic models: homozygous model, OR = 0.62, 95% CI = 0.49–0.77; heterozygous model, OR = 0.71, 95% CI = 0.60–0.84; recessive model, OR = 0.83, 95% CI = 0.76–0.91; dominant model, OR = 0.62, 95% CI = 0.53–0.72; and allele comparison, OR = 0.82, 95% CI = 0.75–0.89.

**Table 2:** Meta-analysis of the association between the ADH1B Arg47His and cancer risk

Variables	Sample size Case/control	Homozygous		Heterozygous		Recessive		Dominant		Allele comparison	
		His/His vs. Arg/Arg		Arg/His vs. Arg/Arg		His/His vs. (Arg/His + Arg/Arg)		(Arg/His + His/His) vs. Arg/Arg		His vs. Arg	
		OR (95% CI)	*P^het^*	OR (95% CI)	*P^het^*	OR (95% CI)	*P^het^*	OR (95% CI)	*P^het^*	OR (95% CI)	*P^het^*
Total	31999/50964	**0.62 (0.49–0.77)**	<0.001	**0.71 (0.60–0.84)**	<0.001	**0.83 (0.76–0.91)**	<0.001	**0.62 (0.53–0.72)**	<0.001	**0.82 (0.75–0.89)**	<0.001
Ethnicity											
Asian	17057/31885	**0.60 (0.48–0.76)**	<0.001	**0.66 (0.53–0.81)**	<0.001	**0.82 (0.75–0.91)**	<0.001	**0.58 (0.47–0.72)**	<0.001	**0.80 (0.72–0.88)**	<0.001
Caucasian	12970/16908	**1.45 (1.05–2.02)**	0.727	1.01 (0.90–1.13)	0.570	**1.45 (1.05–2.00)**	0.712	0.81 (0.64–1.03)	<0.001	1.06 (0.96–1.17)	0.569
Mixed	1972/2171	**0.35 (0.13–0.93)**	0.743	**0.53 (0.37–0.75)**	0.606	**0.37 (0.14–0.98)**	0.751	**0.46 (0.36–0.60)**	0.651	**0.50 (0.36–0.68)**	0.545
Cancer type											
Colorectal	4821/7697	1.19 (0.82–1.72)	<0.001	0.99 (0.88–1.11)	0.857	1.19 (0.91–1.55)	<0.001	1.03 (0.88–1.21)	0.099	1.05 (0.90–1.23)	<0.001
Hepatocellular	1111/3820	0.84 (0.64–1.10)	0.541	1.16 (0.84–1.61)	0.328	0.81 (0.61–1.08)	0.041	0.98 (0.76–1.28)	0.452	0.88 (0.76–1.02)	0.270
Esophageal	9117/15930	**0.39 (0.28–0.55)**	<0.001	**0.47 (0.34–0.64)**	<0.001	**0.72 (0.62–0.83)**	<0.001	**0.41 (0.31–0.54)**	<0.001	**0.67 (0.57–0.78)**	<0.001
Gastric	1770/2930	1.02 (0.76–1.36)	0.637	1.03 (0.84–1.27)	0.356	1.02 (0.86–1.22)	0.973	0.77 (0.48–1.23)	<0.001	1.03 (0.92–1.16)	0.629
Head and neck	6646/7901	**0.55 (0.31–0.97)**	<0.001	0.77 (0.52–1.12)	<0.001	**0.78 (0.66–0.93)**	0.092	**0.64 (0.47–0.87)**	<0.001	**0.80 (0.66–0.96)**	<0.001
UADT	7613/9173	**0.31 (0.23–0.42)**	0.921	**0.33 (0.21–0.53)**	0.161	**0.70 (0.57–0.86)**	0.260	**0.39 (0.26–0.58)**	0.010	**0.62 (0.54–0.71)**	0.924
Pancreatic	395/1865	1.65 (0.62–4.38)	0.345	1.29 (0.76–2.20)	0.430	1.09 (0.78–1.52)	0.513	1.26 (0.75–2.13)	0.358	1.12 (0.86–1.45)	0.774
Control source											
HB	10560/20932	**0.53 (0.40–0.71)**	<0.001	**0.64 (0.51–0.81)**	<0.001	**0.79 (0.69–0.90)**	<0.001	**0.56 (0.44–0.72)**	<0.001	**0.77 (0.68–0.87)**	<0.001
PB	21439/30032	0.75 (0.52–1.07)	<0.001	0.79 (0.61–1.02)	<0.001	0.89 (0.78–1.02)	<0.001	**0.68 (0.54–0.85)**	<0.001	**0.87 (0.76–0.99)**	<0.001
HWE											
YES	20769/37678	**0.60 (0.48–0.76)**	<0.001	**0.71 (0.60–0.84)**	<0.001	**0.81 (0.74–0.89)**	<0.001	**0.67 (0.56–0.81)**	<0.001	**0.81 (0.74–0.88)**	<0.001
NO	1987/1892	0.75 (0.22–2.65)	<0.001	0.66 (0.23–1.90)	<0.001	1.08 (0.76–1.55)	0.006	0.72 (0.25–2.09)	<0.001	0.92 (0.59–1.45)	<0.001

Abbreviations: HB, hospital-based; PB, population-based; UADT, upper aerodigestive tract.

Values in bold indicate *P*<0.05.

Regarding the stratified analysis by ethnicity, a decreased cancer risk was also detected amongst Asians under all the genetic models: homozygous model, OR = 0.60, 95% CI = 0.48–0.76; heterozygous model, OR = 0.66, 95% CI = 0.53–0.81; recessive model, OR = 0.82, 95% CI = 0.75–0.91; dominant model, OR = 0.58, 95% CI = 0.47–0.72; and allele comparison, OR = 0.80, 95% CI = 0.72–0.88, and amongst mixed ethnic group: homozygous model, OR = 0.35, 95% CI = 0.13–0.93; heterozygous model, OR = 0.53, 95% CI = 0.37–0.75; recessive model, OR = 0.37, 95% CI = 0.14–0.98; dominant model, OR = 0.46, 95% CI = 0.36–0.60; and allele comparison, OR = 0.50, 95% CI = 0.36–0.68. However, an increased risk of cancer was detected amongst Caucasians under homozygous model (OR = 1.45, 95% CI = 1.05–2.02) and recessive model (OR = 1.45, 95% CI = 1.05–2.00).

Regarding the stratified analysis by cancer type, the *ADH1B* Arg47His polymorphism significantly decreased the risk of esophageal cancer: homozygous model, OR = 0.39, 95% CI = 0.28–0.55; heterozygous model, OR = 0.47, 95% CI = 0.34–0.66; recessive model, OR = 0.72, 95% CI = 0.62–0.83; dominant model, OR = 0.41, 95% CI = 0.31–0.54; and allele comparison, OR = 0.67, 95% CI = 0.57–0.78; upper aerodigestive tract cancer: homozygous model, OR = 0.31, 95% CI = 0.23–0.42; heterozygous model, OR = 0.33, 95% CI = 0.21–0.53; recessive model, OR = 0.70, 95% CI = 0.57–0.86; dominant model, OR = 0.39, 95% CI = 0.26–0.58; and allele comparison, OR = 0.62, 95% CI = 0.54–0.71; and head and neck cancer: homozygous model, OR = 0.55, 95% CI = 0.31–0.97; recessive model, OR = 0.78, 95% CI = 0.66–0.93; dominant model, OR = 0.64, 95% CI = 0.47–0.87; and allele comparison, OR = 0.80, 95% CI = 0.66–0.96.

Regarding the stratified analysis by control source and HWE, a decreased cancer risk was detected in hospital-based studies: homozygous model, OR = 0.53, 95% CI = 0.40–0.71; heterozygous model, OR = 0.64, 95% CI = 0.51–0.81; recessive model, OR = 0.79, 95% CI = 0.69–0.90; dominant model, OR = 0.56, 95% CI = 0.44–0.72; and allele comparison, OR = 0.77, 95% CI = 0.68–0.87; population-based studies: dominant model, OR = 0.68, 95% CI = 0.54–0.85; and allele comparison, OR = 0.87, 95% CI = 0.76–0.99; and also the studies in agreement with HWE: homozygous model, OR = 0.60, 95% CI = 0.48–0.76; heterozygous model, OR = 0.71, 95% CI = 0.60–0.84; recessive model, OR = 0.81, 95% CI = 0.74–0.89; dominant model, OR = 0.67, 95% CI = 0.56–0.81; and allele comparison, OR = 0.81, 95% CI = 0.74–0.88.

### Sensitivity analysis and publication bias

**Figure 3:**
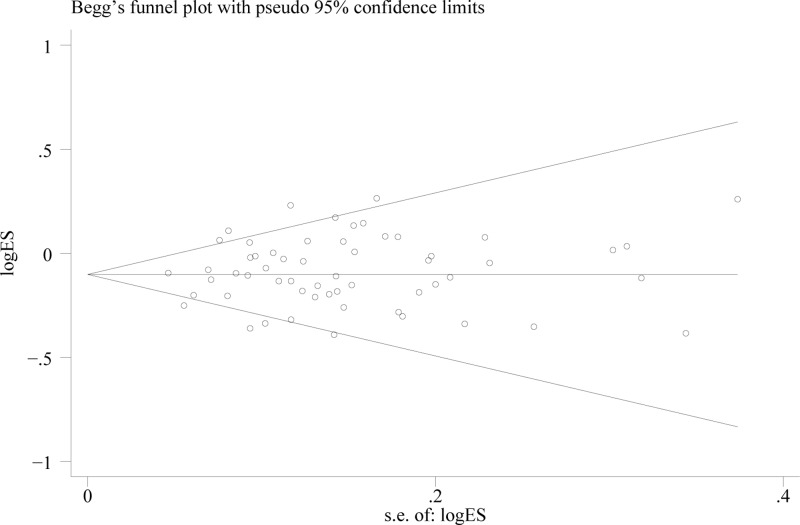
Funnel plot analysis to detect publication bias for *ADH1B* Arg47His polymorphism under the allele comparison model

Substantial heterogeneities were found under all the five genetic models (*P*<0.001). Therefore, the random-effect model was adopted to assess the ORs and 95% CIs. Furthermore, the leave-one-out sensitivity analyses indicated that no single study could change the pooled ORs. The results of the Begg’s funnel plot and Egger’s linear regression test showed no evidence of publication bias (homozygous model, *P*=0.227; heterozygous model, *P*=0.697; recessive model, *P*=0.663; dominant model, *P*=0.599; and allele comparison *P*=0.342, see Figure 3).

## Discussion

Alcohol consumption has been established to be a major factor in the development and progress of cancer [13]. Alcohol is first catalytically oxidized to acetaldehyde, mainly by ADH, and then to harmless acetate by ALDH [6,7]. Acetaldehyde, a Group I human carcinogen classified by the International Agency for Research on Cancer (IARC), may stimulate carcinogenesis by disrupting DNA synthesis and repair [8,9,84]. Therefore, to reduce the risk of cancer, it is important to modulate exposure levels to acetaldehyde in the liver. *ADH1B* gene, also known as *ADH2*, is located on chromosome 4q22 and is the locus responsible for the majority of activities of ADH function [25]. Arg47His (rs1229984 G > A) in *ADH1B* led to a single amino acid substitution of arginine (Arg) for histidine (His) at codon 47. Compared with the Arg/Arg individuals, the His/His individuals have a 40-fold higher enzyme activity oxidized alcohol to toxic acetaldehyde, thereby inducing tumorigenesis [25,85].

To the best of our knowledge, this is the first meta-analysis investigating the association between *ADH1B* Arg47His polymorphism and the overall cancer risk. A total of 66 studies from 64 articles with 31999 cases and 50964 controls were included, and the large sample size provided adequate power to detect this association. Overall, *ADH1B* Arg47His polymorphism was associated with a decreased risk of overall cancer under all the five genetic models. Stratified analysis by ethnicity revealed that *ADH1B* Arg47His polymorphism reduced cancer risk amongst Asians and mixed ethnicity group but increased risk amongst Caucasians. Stratified analysis by cancer type revealed that *ADH1B* Arg47His polymorphism reduced risk in esophageal cancer, upper aerodigestive tract cancer, and head and neck cancer, while no effect was found on colorectal, hepatocellular, gastric and pancreatic cancer. In stratified analysis by control source and HWE, a decreased cancer risk was detected in hospital-based studies, population-based studies, and also the studies in agreement with HWE.

There were several meta-analyses focussed on *ADH1B* Arg47His polymorphism and only one particular type of cancer risk, such as esophageal, head and neck, gastric and colorectal cancer [11–15]. For esophageal cancer, Mao et al. [11] found that the 47His allele was significantly associated with the reduced risk of this cancer when compared with the 47Arg allele. And these findings were replicated in our meta-analysis. For head and neck cancer, the 47His allele was also found to be associated with decreased risk of head and neck cancer amongst Asians only under the dominant model [12]. However, similar results were found under the other three models in our analysis, which may be attributed to a larger sample size including eight more studies. Interestingly, Chen et al. [15] found that *ADH1B* Arg47His polymorphism was associated with decreased risk of colorectal cancer supported by four studies. However, this decreased risk was not present in the current one including six more studies. It was noteworthy that we found that *ADH1B* Arg47His polymorphism was associated with decreased cancer risk amongst Asians while increased cancer risk amongst Caucasians. In Caucasian population, the A allele was found to associate with an increased risk of colorectal cancer [32]. The opposite findings may result from the difference of ethnicity with the 47His allele occupied more than 90% amongst Asians but fewer than 20% amongst Caucasians [7]. Furthermore, we re-analyzed the ethnic groups of Asian and Caucasian people. Amongst Asians, a decreased cancer risk was also detected in esophageal cancer and head and neck cancer. While in Caucasians, we did not repeat the results, but an increased cancer risk was detected in colorectal cancer (homozygous model, OR = 1.55, 95% CI = 1.10–2.20 and recessive model, OR = 1.55, 95% CI = 1.11–2.18).

Several limitations in the current meta-analysis should be addressed. First, a number of studies adopted in our meta-analysis had relatively small sample size for each cancer type, like bladder and breast cancer. Second, because of the absence of original data, our analyses were based on unadjusted estimates of ORs without adjustment for other confounding factors. Third, there were substantial heterogeneities in all the five genetic models, hence the random-effect model was adopted and might present unstable results. Overall, due to these limitations, the findings in the current meta-analysis should be interpreted with caution.

In conclusion, our meta-analysis suggested that *ADH1B* Arg47His polymorphism was significantly associated with the decreased overall cancer risk, especially for esophageal cancer and head and neck cancer amongst Asians.

## Abbreviations

ADH: alcohol dehydrogenase
ALDH: aldehyde dehydrogenase
CI: confidence interval
HWE: Hardy–Weinberg equilibrium
OR: odds ratio
